# Fundamentos da Pericardite Aguda: Estado da Arte

**DOI:** 10.36660/abc.20240572

**Published:** 2026-03-26

**Authors:** Gabriel de Moraes Mangas, Jose Gregorio Valero Rodriguez, João Alexandre Ranzeiro de Bragança dos Santos, Flavia Nunes Benicio de Souza, Luis Felipe Leite da Silva, Giovane Leal de Azevedo, Alberto Chivaca, Neusa Perina de Jesus Jessen, Ângelo Oliveira, Evandro Tinoco Mesquita

**Affiliations:** 1 Universidade Federal Fluminense Niterói RJ Brasil Universidade Federal Fluminense, Niterói, RJ – Brasil; 2 Hospital Central de Maputo Maputo Moçambique Hospital Central de Maputo, Maputo – Moçambique; 3 Universidade Eduardo Mondlane Maputo Moçambique Universidade Eduardo Mondlane, Maputo – Moçambique; 4 Complexo Hospitalar de Niterói Niterói RJ Brasil Complexo Hospitalar de Niterói, Niterói, RJ – Brasil

**Keywords:** Pericardite Aguda, Iinflamação, Diagnóstico, Tratamento, Inteligência Artificial

## Abstract

A pericardite aguda é uma inflamação do pericárdio, o revestimento que envolve o coração, sendo a condição inflamatória cardíaca mais comum. A doença afeta frequentemente adultos jovens e pode se manifestar com gravidade variável. A pericardite pode ter diversas causas, incluindo infecções virais, doenças autoimunes, condições pós-infarto e, recentemente, infecções por SARS-CoV-2 ou vacinas contra a COVID-19. Nos países em desenvolvimento, especialmente na África, a tuberculose é a principal causa de pericardite, frequentemente associada ao HIV. O diagnóstico baseia-se em critérios clínicos, como dor no peito e alterações no eletrocardiograma e pode ser apoiado por exames de imagem, como ecocardiograma, ressonância magnética cardíaca e tomografia computadorizada. A identificação da etiologia é crucial para o tratamento personalizado, embora, muitas vezes, a causa específica não seja identificada. Novas pesquisas têm destacado o papel do inflamassoma NLRP3 na fisiopatologia da pericardite, o que pode abrir caminho para novas terapias. Além disso, avanços tecnológicos e a inteligência artificial são discutidos como ferramentas promissoras para melhorar o manejo da pericardite.

## Introdução

A pericardite aguda é a patologia inflamatória cardíaca mais comum, com uma incidência estimada em 27,7 a cada 100.000 pessoas no mundo Ocidental.^1^ No continente africano, uma revisão sistemática da evidência existente mostrou que a prevalência de doenças pericárdicas varia muito de acordo com a população estudada, tendo sido encontradas prevalências de cerca de 1,1% em indivíduos com queixas cardíacas, entre 3,3% e 6,8% em pacientes com insuficiência cardíaca e até 46,5% em pacientes infectados pelo HIV e com sintomas cardíacos.^2^

Embora geralmente siga um curso benigno e autolimitado, a pericardite pode apresentar-se com quadros graves. A condição frequentemente afeta adultos jovens, com recorrência comum.^3,4^ O prognóstico está associado à idade do paciente e à etiologia da pericardite.^5,6^ Diante disso, uma abordagem multidisciplinar, envolvendo uma colaboração entre cardiologistas, reumatologistas, oncologistas, imunologistas e infectologistas, pode resultar em melhores desfechos para os pacientes.^7,8^ Os avanços no entendimento fisiopatológico da pericardite aguda revelaram o papel central do inflamassoma NLRP3 e da subsequente liberação de interleucinas (IL-1α e IL-1β) no desenvolvimento da resposta inflamatória.^9,10^ Esse processo fisiopatológico tem sido alvo de novas abordagens terapêuticas para a pericardite, como o antagonista do receptor IL-1, rilonacept.^11^

Nesse contexto, a presente revisão visa destacar os recentes avanços no tratamento, diagnóstico, fatores prognósticos, fisiopatologia e as atuais diretrizes acerca da pericardite aguda. Ademais, será discutido o potencial papel de novas tecnologias, por meio da inteligência artificial, no desenvolvimento de uma melhor conduta em relação aos pacientes com pericardite.

## Métodos

Foi realizada uma revisão narrativa sobre pericardite, conduzida por meio de uma busca eletrônica estruturada. A estratégia de busca foi delineada com o uso de descritores controlados (MeSH/DeCS) combinados por operadores booleanos (AND, OR), abrangendo estudos publicados em inglês, português ou espanhol no período de janeiro de 2010 a agosto de 2024. As buscas foram realizadas nas bases de dados MEDLINE/PubMed, Cochrane Library, LILACS/IBECS e Embase, e incluídos estudos primários, relatos de casos, revisões sistemáticas, revisões narrativas e estudos qualitativos. Adicionalmente, foram identificados artigos relevantes por meio da revisão das referências bibliográficas dos estudos selecionados e da busca complementar na web, a fim de captar publicações não localizadas na busca inicial. Foram excluídos aqueles que não abordaram o diagnóstico ou o manejamento de pericardite. Os termos de busca utilizados incluíram: "Pericarditis"; "Pericardite"; "Acute Pericarditis"; "Pericardite Aguda"; "Inflammation"; "Inflamação"; "Diagnosis"; "Diagnóstico"; "Diagnostic Imaging"; "Imagem Diagnóstica"; "Therapeutics"; "Tratamento"; "Treatment"; "Anti-inflammatory agents"; "Follow-Up Studies"; "Acompanhamento"; "Monitoring"; "Long-term follow-up"; "Artificial Intelligence"; "Inteligência Artificial"; "Machine Learning"; "Aprendizado de Máquina". Dada a natureza do estudo como uma revisão narrativa, sem envolvimento direto de seres humanos ou animais, não foi necessária a aprovação por um comitê de ética.

**Figure:**
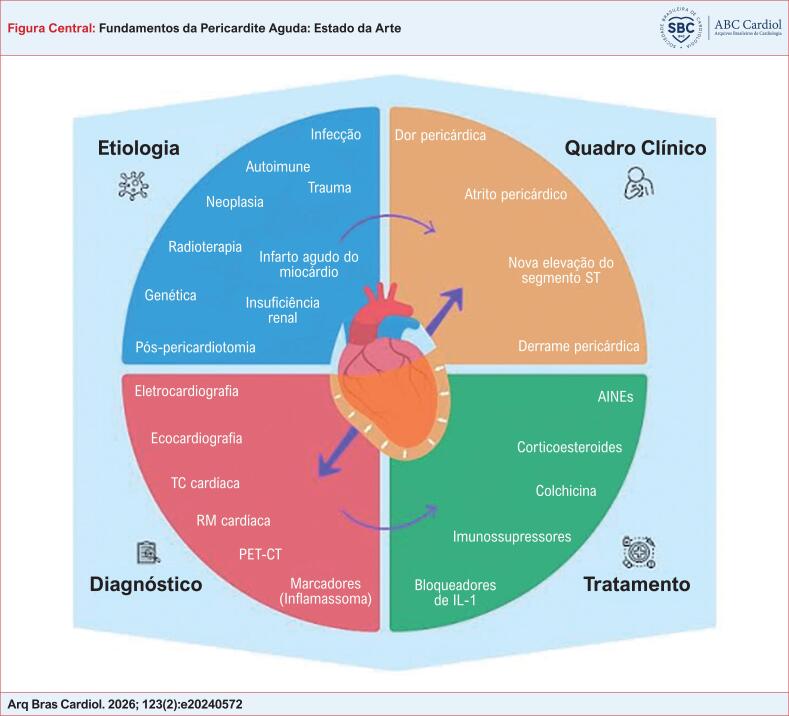
Mapa conceitual sobre etiologia, quadro clínico, diagnóstico e tratamento medicamentoso da pericardite aguda. AINEs: anti-inflamatórios não esteroidais; IL-1: interleucina-1; PET-CT: tomografia por emissão de pósitrons associada à tomografia computadorizada: RM: ressonância magnética; TC: tomografia computadorizada.

### Etiofisiopatologia

O saco pericárdico é composto por duas camadas: a visceral e a parietal. A camada visceral, composta por células mesoteliais pericárdicas, adere diretamente ao epicárdio, enquanto a camada parietal, mais fibrosa, fornece suporte estrutural. As células mesoteliais pericárdicas são essenciais para o funcionamento adequado do coração. Elas desempenham um papel crucial na lubrificação, na proteção e na regulação da inflamação no pericárdio. Alterações na função das células mesoteliais podem levar a diversas condições, como pericardite, derrame pericárdico e tamponamento cardíaco. Entretanto, a dor referida no ombro é devido às fibras somatossensoriais do nervo frênico que inervam o pericárdio parietal.^11^

A pericardite é a forma mais comum de doença do pericárdio e é definida como a inflamação das camadas do pericárdio. Ela pode estar associada ao derrame pericárdico e resultar em tamponamento cardíaco. Essa doença pode ser uma manifestação cardíaca isolada ou resultado de uma desordem sistêmica.

A pericardite pode ter diversas etiologias, tanto infecciosas quanto não infecciosas, ou ser idiopática (Figura Central).^12^ Como ilustrado na Tabela 1, a pericardite pode ser causada por infecção (viral, bacteriana, micótica e parasitária), doenças autoimunes, doenças metabólicas, pós-infarto do miocárdio, trauma, síndrome pós-pericardiotomia, neoplasias, pós-radioterapia e medicamentos.^13^ Dentre as etiologias, as mais comuns são as causas infecciosas, predominando a pericardite viral (80% a 85% dos casos aproximadamente).^1^ Nesse caso, a inflamação decorre da ação direta do vírus ou da resposta imune do organismo a esse agente.^14^ Outrossim, quadro clínico de aparente infecção viral recente (infecção do trato respiratório superior ou gastroenterite) foi documentado em aproximadamente 40% dos pacientes com pericardite aguda.^1^

**Tabela 1:** Principais agentes etiológicos da pericardite aguda

Etiologia	Detalhamento	Observações importantes
**Infecciosa**	Viral, bacteriana, micótica e parasitária	– 80% a 85% de todos os casos são virais. – Cerca de 40% dos pacientes com pericardite aguda têm história de infecção recente (via aérea superior ou gastrointestinal). – Os casos ocasionados por infecção bacteriana podem ter origem de pneumonia, febre reumática, empiema, disseminação hematogênica, pós cirurgia cardíaca/torácica. – Em países em desenvolvimento, 70% a 80% dos casos é devido à tuberculose. – Infecção pelo HIV é fator de risco.
**Doença autoimune**	Lúpus eritematoso sistêmico, artrite reumatoide, esclerodermia, polimiosite e dermatomiosite	
**Doença metabólica**	Hipotireoidismo, hiperlipidemia e diabetes mellitus	Afetam de forma indireta o pericárdio por meio de reações inflamatórias ou metabólitos tóxicos às células do pericárdio.
**Infarto agudo do miocárdio**	Reação inflamatória	Pode ocorrer precoce (3 dias pós), denominada pericardite epistenocárdica, ou tardiamente (3 semanas a 6 meses) devido à autoimunidade, sendo denominada síndrome de Dressler.
**Trauma**	Objeto externo	Principalmente em tórax
**Síndrome pós-pericardiotomia**	Reação inflamatória local ou sistêmica	Tem se tornado mais comum com aumento das intervenções.
**Neoplasias**	Invasão tumoral ou linfática	Disseminação hematogênica
**Radioterapia**	Danos direto ao tecido (células do pericárdio), reação inflamatória, fibrose e dano vascular	Principalmente em câncer torácico, especialmente em câncer de mama, pulmão e linfoma
**Medicamentoso**	Cardiotoxicidade devido à radioterapia e quimioterapia	Inibidores de imuno-checkpoints são drogas de uso recente que podem estar associadas à pericardite.
**Insuficiência renal**	Pericardite urêmica ou associada à diálise	Cerca de 20% evoluem para derrame pericárdico.

Ademais, para a definição da etiologia, dados epidemiológicos locais devem sempre ser considerados.^1^ Com base em evidências que existem em países desenvolvidos, os agentes etiológicos mais prevalentes são os vírus e, com frequência, os casos de pericardite aguda são precedidos por uma síndrome gripal ou gastrointestinal. Observa-se, também, aumento dos casos de pericardite aguda durante as estações mais frias. Em um estudo prospectivo da França, 55% dos casos foram de origem idiopática e 20% após síndrome de injúria cardíaca. Essa última causa está aumentando progressivamente em países desenvolvidos devido ao crescente número de procedimentos cardíacos.^12^ A pericardite pós-infarto pode ocorrer precocemente, nos 3 primeiros dias após o episódio de infarto agudo do miocárdio, sendo denominada pericardite epistenocárdica, ou tardiamente – de 3 semanas a 6 meses – devido à atividade autoimune, que recebe o nome de síndrome de Dressler.

Outrossim, a pericardite em pessoas vivendo com HIV pode ser devida a doenças infecciosas, não infecciosas ou neoplásicas (sarcoma de Kaposi ou linfoma), podendo resultar em miopericardite. E nos casos de etiologia autoimune, a pericardite acontece principalmente nos casos de lúpus eritematoso sistêmico, artrite reumatoide, esclerodermia, polimiosite e dermatomiosite. Outra causa comum de doença pericárdica é a insuficiência renal, a qual evolui com o desenvolvimento de derrame em 20% dos casos, podendo manifestar-se como pericardite urêmica ou pericardite associada a diálise.^14^

Outras possíveis causas a serem consideradas são hipotireoidismo e câncer. No caso das pericardites neoplásicas, elas são consequência da invasão tumoral ou linfática, ou ocorrem por disseminação hematogênica, ocorrendo, então, tanto na forma primária quanto na metastática, ou mesmo como resultado de radioterapia para cânceres torácicos. Atualmente, a pericardite por inibidores de imuno-checkpoints tem alguns poucos casos descritos na literatura.^3,12^ Outro mecanismo associado à pericardite aguda em um paciente oncológico envolve a cardiotoxicidade decorrente de medicamentos radioterápicos e mais recentemente inibidores de imuno-checkpoints.^12^

Entretanto, quando de etiologia bacteriana, normalmente, a pericardite cursa com derrame pericárdico, e sua origem pode derivar de situações como pneumonia, febre reumática, empiema, disseminação hematogênica, pós-cirurgia cardíaca ou torácica.^14^

Nos países em desenvolvimento, a elevada prevalência de doenças infecciosas como o HIV, associada à pobreza e aos fracos sistemas de saúde existentes contribui para que as doenças infecciosas tenham um papel importante no espectro da etiologia da insuficiência cardíaca. Numa análise de estudos que avaliaram a etiologia da insuficiência cardíaca em vários países da África subsaariana, a pericardite foi encontrada em 3% dos casos.^15^ No estudo The Sub-Saharan Africa Survey of Heart Failure (THESUS–HF) incluindo 1.006 pacientes com insuficiência cardíaca de 9 países africanos, 6,8% apresentaram derrame pericárdico com tamponamento.^16^ Ressalta-se ainda que, em países em desenvolvimento, a principal etiologia da pericardite é a tuberculose, alcançando 70% a 80% dos casos ou 90% entre os pacientes HIV positivos.^17^

Em África em particular, uma revisão sistemática das evidências disponíveis mostrou que poucos estudos reportaram sobre a etiologia da doença pericárdica e a maior parte deles incluiu uma amostra pequena de pacientes.^18^ Entre os estudos publicados, a tuberculose foi a causa de doença pericárdica mais frequentemente observada e a infecção pelo HIV foi o principal fator de risco; entretanto, a tuberculose foi a principal etiologia também em pacientes não infectados pelo HIV. Em um estudo de base ecocardiográfica realizado num hospital terciário de Dar es Salaam, foi avaliado o padrão de anomalias cardíacas encontradas em doentes infectados pelo HIV e que se apresentaram com sintomas cardíacos. O diagnóstico ecocardiográfico mais comum foi derrame pericárdico, presente em 41,2% dos participantes do estudo.^19^ No entanto, em grande parte dos casos reportados, a etiologia da doença pericárdica não foi encontrada e nos restantes casos as neoplasias e doenças sistêmicas como o lúpus eritematoso sistêmico foram as etiologias mais encontradas.^18^

Além das mais conhecidas etiologias da pericardite aguda, duas novas importantes etiologias têm sido descritas nos últimos 2 anos. São elas a infecção por SARS-CoV-2 e a vacina contra a COVID-19, especialmente a vacina de RNAm. Com essas duas novas etiologias, a pericardite que cursa com miocardite tem ganhado mais relevância. A pericardite esteve presente em 1,5% dos casos de COVID-19 e é mais comum em indivíduos mais velhos. Embora seja uma etiologia possível de pericardite, os dados atuais evidenciam que os benefícios da vacinação contra as infecções respiratórias contrabalançam preocupações sobre a possível, mas definitivamente rara, pericardite induzida por vacinação. Ademais, o curso clínico da pericardite por essa etiologia não se difere quando comparado ao quadro comum de pericardite viral.^20,21^

No período do diagnóstico da pericardite, a infecção viral não pode ser confirmada pela sorologia de anticorpos, já que os anticorpos IgM não são mais detectados. Técnicas moleculares, como Reação em Cadeia da Polimerase (PCR), no fluido pericárdico ou no tecido depois de uma pericardiocentese ou de uma biópsia pericárdica, respectivamente, poderia ser capaz de identificar o agente etiológico, mas é uma abordagem invasiva e não recomendada para uma desordem que geralmente segue um curso benigno. Segundo diretrizes recentes, a identificação rotineira do agente viral não é recomendada já que não afeta as decisões de tratamento e o prognóstico, com exceção do vírus da hepatite C e do HIV. Os termos pericardite viral e idiopática são tratados como sinônimos, assumindo-se etiologia viral presumida na forma idiopática. Em suma, a identificação da etiologia subjacente é de suma importância para o desenvolvimento de um plano personalizado de tratamento da pericardite, sendo recomendável que se pesquise a presença de pericardite em pacientes que apresentem doenças sistêmicas.^1^

Em relação à fisiopatologia molecular e celular, novos ensaios clínicos em roedores têm mostrado que o inflamassoma NLRP1 (domínio NACHT, repetição rica em leucina e pirina 1) e a IL-1β possuem importante papel no desenvolvimento da inflamação da pericardite aguda e em sua recorrência.^22^ Os inflamassomas são plataformas compostas por proteínas que controlam a resposta inflamatória e coordenam as defesas antimicrobianas do organismo. Eles são ativados por meio da detecção de microrganismos patogênicos e sinais de perigo no citoplasma das células, ativando a caspase-1, que produz citocinas e induz a piroptose das células, por meio da ativação da gasdermina D. Os inflamassomas, em sua ativação exacerbada, estão relacionados com numerosas desordens inflamatórias hereditárias e adquiridas, como a pericardite.^13^ Essas evidências devem guiar os próximos estudos que visam investigar o mecanismo de desenvolvimento da pericardite que, por muitas vezes, não possui etiologia definida, sendo denominada idiopática.^22^

### Diagnóstico

O diagnóstico da pericardite aguda deve incluir dois dos seguintes quadros: dor pericárdica, atrito pericárdico, nova elevação do segmento ST ou depressão do segmento PR no eletrocardiograma (ECG), ou o aparecimento de novo ou o agravamento de um derrame pericárdico (demonstrado por um método de cardioimagem) (Figura Central).^12^ A dor pericárdica é aguda, retrosternal e, tipicamente, piora com a inspiração ou tosse e com o decúbito e melhora quando o paciente se senta ereto ou inclinado para frente.^11^ Ela pode ainda se irradiar para o topo das escápulas, braços, pescoço ou mandíbula.^12,23^ No exame físico, além do atrito pericárdico, que pode ser auscultado na borda esternal esquerda, podem ser encontradas febre baixa e outras manifestações, caso a doença de base aja sistemicamente. Não existe biomarcador específico para pericardite; no entanto, biomarcadores de inflamação, como proteína C-reativa, estão aumentados em 80% dos casos e a troponina pode estar levemente elevada, caso a inflamação tenha atingido o miocárdio – quadro que passa a ser chamado miopericardite.^12^

Por outro lado, o diagnóstico etiológico é um desafio e em muitos casos não se encontra a causa da pericardite. Inclusive no caso específico de suspeita de pericardite tuberculosa, o diagnóstico definitivo tem sido difícil, o que levou ao estudo da acurácia de novos testes de diagnóstico incluindo o teste de PCR quantitativo (Xpert MTB/RIF), o interferon gama não estimulado (uIFNγ), a adenosina desaminase pericárdica (ADA) e o lipoarabinomanano pericárdico e urinário (LAM). Em um estudo comparativo entre o teste Xpert MTB/RIF, ADA pericárdica e uIFNγ, o ensaio uIFNγ apresentou maior precisão diagnóstica podendo ser o teste de primeira linha para o diagnóstico de pericardite tuberculosa. Adicionalmente, maior sensibilidade e especificidade é conseguida com a combinação de Xpert MTB/RIF com ADA ou uIFNγ.^24^ Por outro lado, a LAM urinária ou pericárdica mostrou baixa sensibilidade, mas alta especificidade para o diagnóstico de pericardite tuberculosa e a LAM urinária mostrou maior sensibilidade em pacientes infectados pelo HIV com CD4 ≤ 100 células/mm^3^.^25^ No entanto, o acesso a estes testes diagnósticos apresenta-se como outro desafio importante nos países africanos, onde a entidade assume maior importância na etiologia das pericardites. Assim sendo, quando se consegue colher o líquido pericárdico, a cultura do líquido continua sendo o teste diagnóstico mais utilizado e a ADA continua sendo também o teste bioquímico mais utilizado. No entanto, a cultura leva pelo menos 3 semanas para produzir resultados e a ADA tem limitado valor como teste confirmatório de diagnóstico. Além disso, nestas áreas de alta endemicidade para tuberculose, o teste cutâneo de tuberculina positivo apenas reflete a exposição aos antígenos do *Mycobacterium tuberculosis*, não discernindo com precisão a doença ativa, e por isso tem valor limitado.^26^

**Figura 1:**
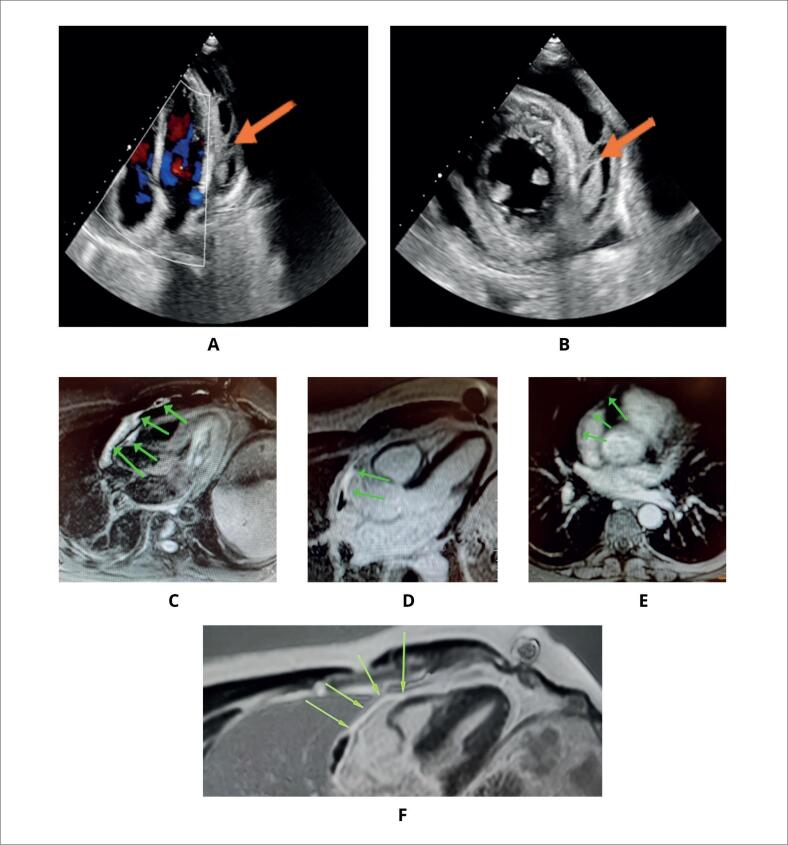
A, B) Ecocardiograma de janela apical de 4 câmaras e janela paraesternal eixo curto, respectivamente, mostrando a presença de derrame e bastante fibrina no espaço pericárdico (setas). C, D, E, F) Ressonância magnética mostrando extensa fibrose de padrão não isquêmico em regiões mesocárdica e subepicárdica.

Exames de imagem podem ser úteis no diagnóstico e acompanhamento da pericardite aguda, especialmente ecocardiografia, ressonância magnética cardíaca (RMC) e tomografia computadorizada (TC) (Figura 1).^23^ Na ausência de uma efusão pericárdica de volume suficiente, a radiografia convencional normalmente não apresenta alterações.^11^

**Figura 2:**
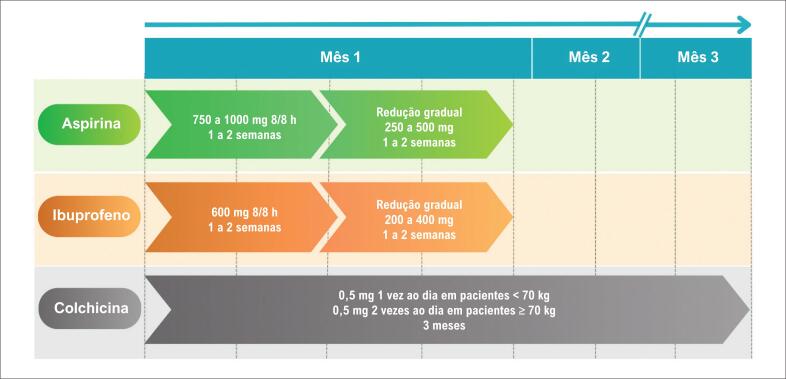
Esquema de tratamento medicamentoso da pericardite.

A ecocardiografia deve ser o exame de imagem de primeira escolha, por ser um método acessível, de baixo custo e de rápida execução, capaz de fornecer informações fundamentais sobre a espessura do pericárdio, a presença e o volume do derrame pericárdico, bem como seu impacto hemodinâmico no enchimento cardíaco (tamponamento pericárdico). O exame também pode identificar massas aderidas à cavidade pericárdica, trabéculas de fibrina, conteúdo com densidade sugestiva de trombo no líquido pericárdico e formas localizadas de derrame, como frequentemente observado no pós-operatório de cirurgia cardíaca (Figura 2). O ecocardiograma pode ser normal trazendo como diagnóstico uma pericardite seca, que pode estar presente em 30% dos pacientes. Adicionalmente, pequenos derrames pericárdicos podem acontecer em indivíduos saudáveis; portanto, o diagnóstico de pericardite deve ser feito com cautela. Apesar de alguns pacientes com pericardite aguda apresentarem ecocardiograma sem alterações, esse exame é muito útil para diagnosticar presença de efusão pericárdica e seu tamanho, fisiologia do tamponamento e envolvimento do miocárdio, como numa miopericardite.^17^

A pericardite tuberculosa, por exemplo, apresenta-se geralmente com derrame, muitas vezes em tamponamento, e por vezes já em fase de pericardite constritiva, podendo também apresentar-se em forma de miopericardite (Tabela 1). O importante estudo Investigation of the Management of Pericarditis in Africa (IMPI Africa) recrutou 185 pacientes de 15 hospitais de três países da África Subsaariana que estavam no início do tratamento para pericardite tuberculosa. Destes, 79,5% apresentavam pericardite efusiva, 15,1% efusiva-constritiva e 5,4% constritiva ou aguda seca, e 40% dos pacientes apresentavam características clínicas de infecção pelo HIV.^27^

Se os resultados da ecocardiografia forem inconclusivos ou houver apresentação clínica atípica, ou se o médico quiser investigar causas específicas, como malignidade ou tuberculose, TC ou RMC podem ser utilizadas. Na TC podemos observar a espessura, calcificação, derrames pericárdicos regionais e, através da densidade do líquido pericárdico, podemos diferenciar se é um transudato, exsudato ou sangue e, ocasionalmente, a presença de massa próxima ao pericárdio, como tumores de pulmão e mama. Um recurso muito útil na RMC é o realce tardio de gadolínio (RTG), uma técnica que permite a detecção de inflamação pericárdica com sensibilidade de 94% a 100%.^12,17^

O PET-CT com 18F-FDG é um exame mais comumente utilizado no contexto da oncologia, no entanto, provou-se útil, na medida em que, no contexto de pericardite aguda com efusão pericárdica, uma maior absorção de FDG pelo pericárdio está associada a maior risco de recorrência e pode ser útil na caracterização da pericardite recorrente.^17^

### Diagnóstico diferencial

O diagnóstico diferencial da pericardite aguda é essencial devido à sua sobreposição de sintomas com várias condições cardíacas e não cardíacas. Dor torácica súbita é um sintoma comum, mas não específico, e pode ser causada por diversas condições, incluindo infarto do miocárdio, embolia pulmonar, dissecção aórtica, além de distúrbios gastrointestinais e musculoesqueléticos. A avaliação inicial inclui exames físicos detalhados e análise dos sintomas do paciente. Exames laboratoriais, como dosagem de troponina e proteína C-reativa, são úteis para diferenciar a pericardite aguda de um infarto do miocárdio.^12,13^ O ECG frequentemente revela elevação difusa do segmento ST, infra de PR e os estágios eletrocardiográficos da pericardite aguda, um achado clássico, embora não específico, da doença. A pericardite, classicamente, está associada a alterações no ECG que evoluem em quatro estágios (Tabela 2).^28^

**Tabela 2:** Alterações no ECG conforme progressão da pericardite

Estágios	Alterações no ECG
**Estágio 1**	Elevação difusa do segmento ST e depressão do segmento PR com alterações recíprocas na derivação aVR (ocorre durante as primeiras 2 semanas)
**Estágio 2**	Normalização das alterações do segmento ST; achatamento generalizado da onda T (1 a 3 semanas)
**Estágio 3**	As ondas T achatadas tornam-se invertidas (3 a várias semanas)
**Estágio 4**	O ECG volta ao normal (várias semanas em diante)

ECG: eletrocardiograma.

A radiografia de tórax pode mostrar aumento do tamanho do coração ou derrame pleural associado. A ecocardiografia é uma ferramenta valiosa para avaliar a presença de derrame pericárdico e de alterações na função cardíaca, mas pode não ser conclusiva por si só. Em casos de suspeita persistente ou quando o diagnóstico não é claro, a RMC ou a TC pode ser útil para avaliar as estruturas cardíacas com mais detalhes. A exclusão do infarto do miocárdio é um dos principais desafios no diagnóstico diferencial. Em pacientes com suspeita de pericardite aguda e fatores de risco para doença arterial coronariana, pode ser necessária a realização de angiografia coronariana para descartar obstruções coronárias.

Ademais, é importante considerar o diagnóstico diferencial de necrose da gordura epipericárdica (EFN). Assim como na pericardite aguda, a EFN pode apresentar dor torácica como sintoma principal, o que pode dificultar o diagnóstico preciso. Dessa forma, é importante avaliar as diferenças na localização da dor, visto que na NFE é geralmente mais pleurítica e localizada na região precordial, enquanto na pericardite a dor pode ser mais difusa e se irradiar para o pescoço, ombros e costas, com alterações no ECG. A EFN geralmente não causa alterações no ECG, porém a pericardite pode apresentar alterações como elevação do segmento ST e depressão do PR, além de febre, fadiga e atrito pericárdico, que são menos frequentes na NFE. A TC é o exame de escolha para diagnosticar a EFN, pois evidencia a área de inflamação da gordura ao redor do coração. Contudo, a RM e exames laboratoriais podem auxiliar também.^29,30^

É importante considerar outras condições que podem mimetizar a pericardite aguda, como miocardite, embolia pulmonar e doenças gastrointestinais. Cada uma dessas condições apresenta características clínicas distintas e exames específicos que auxiliam no diagnóstico diferencial preciso. A avaliação diagnóstica cuidadosa e a consideração de uma variedade de condições que podem se manifestar de forma semelhante à pericardite aguda são cruciais para um manejo clínico eficaz e preciso. A integração de exames clínicos, laboratoriais e de imagem desempenha papel fundamental na abordagem diagnóstica diferencial desses casos.

Por fim, o uso de critérios diagnósticos inadequados pode levar ao sobrediagnóstico da pericardite aguda. O derrame do pericárdio em pequenos volumes, a dor torácica e os biomarcadores elevados podem ser encontrados em pessoas hígidas, não indicando necessariamente a presença de uma doença. O sobrediagnóstico da pericardite aguda pode levar a tratamentos desnecessários, como a administração de anti-inflamatórios ou corticosteroides, que podem prejudicar a qualidade de vida do paciente, além de elevar o custo para o sistema de saúde.^12,13^

### Tratamento

As principais diretrizes sobre pericardite – ESC (2015), ACC/AHA (2020) e SBC (2013) – apresentam semelhanças no tratamento de primeira linha, mas divergem em aspectos importantes da abordagem e da estratificação de risco (Tabela 3).^14,31,32^

**Tabela 3:** Comparação entre as diretrizes ESC (2015), ACC/AHA (2020) e SBC (2013) para o tratamento da pericardite aguda

Aspecto	ESC (2015)	ACC/AHA (2020)	SBC (2013)
**AINEs**	Ibuprofeno 600 a 800 mg 3 vezes/dia ou aspirina 750 a 1000 mg 3 vezes/dia por 1 a 2 semanas, com desmame gradual	Ibuprofeno, aspirina ou indometacina por 2 a 4 semanas, com desmame gradual após remissão dos sintomas	Ibuprofeno 600 mg 3 vezes/dia ou aspirina 1000 mg 3 vezes/dia por 1 a 2 semanas, com redução progressiva
**Colchicina** **(episódio agudo)**	0,5 a 1,0 mg/dia por 3 meses, ajustada por peso e tolerância	0,5 a 1,0 mg/dia por 3 meses, com ajuste de dose por peso	Recomendação classe IIa, evidência B. Dose: 1 mg/dia (0,5 mg 2 vezes/dia) por 3 meses. Não indicada universalmente
**Colchicina (recorrente)**	0,5 a 1,0 mg/dia por 6 meses	0,5 a 1,0 mg/dia por 6 meses, com ajuste por peso	Recomendada por 6 meses em casos de pericardite recorrente
**Evitar atividade física**	Não especificado	Recomendado evitar esforço físico durante o tratamento	Recomendado repouso clínico, sem definição objetiva de tempo ou intensidade
**Corticoide – indicação**	Reservado para contraindicação ou falha a AINEs/colchicina, pericardite autoimune ou urêmica	Reservado para contraindicação ou falha a AINEs/colchicina, ou pericardite associada a doenças específicas	Recomendado em casos refratários a AINEs/colchicina, pericardite autoimune ou urêmica
**Corticoide – dose e tempo**	Prednisona 0,2 a 0,5 mg/kg/dia, com desmame lento e uso conjunto com colchicina	Prednisona ou equivalente em dose baixa (0,2 a 0,5 mg/kg/dia), com retirada gradual e associação à colchicina	Prednisona 1 mg/kg/dia por 2 semanas, com desmame em 4 a 6 semanas e associação à colchicina
**Risco de recorrência com corticoide**	Uso precoce e isolado associado a maior risco de recorrência	Uso precoce e isolado associado a maior risco de recorrência	Uso isolado pode aumentar risco de recorrência; recomenda-se evitar
**Imunobiológicos (inibidores de IL-1)**	Não especificado	Recomendados em casos refratários ou corticodependentes (ex.: anakinra, rilonacept)	Não mencionados
**Critérios de internação**	Considera fatores como febre, derrame volumoso, e miopericardite como de pior prognóstico	Define critérios objetivos: febre > 38 °C, derrame volumoso, falha terapêutica após 7 dias, miocardite	Indica internação em casos de tamponamento, febre alta, derrame importante ou etiologia grave

AINEs: anti-inflamatórios não esteroidais; IL-1: interleucina-1.

As três orientações recomendam o uso inicial de anti-inflamatórios não esteroidais (AINEs) associados à colchicina. A ESC propõe o uso de AINEs por 1 a 2 semanas, com desmame gradual, e colchicina por 3 meses no primeiro episódio e 6 meses em recidivas, na dose habitual de 0,5 a 1,0 mg/dia, ajustada conforme o peso e a tolerância (Figura 2). A ACC/AHA reforça o mesmo esquema de colchicina, com ajuste por peso corporal, e enfatiza a evitação de atividade física intensa durante o tratamento. Ambas as diretrizes de orientação e os artigos recentes destacam que a adição precoce da colchicina reduz significativamente a taxa de recorrência e previne complicações.^31–34^

O uso de corticosteroides, como prednisona ou prednisolona, é reservado, segundo as três diretrizes, para casos com contraindicação ou falha no uso de AINEs e colchicina, ou para pericardite de etiologia autoimune ou urêmica. A ESC e a ACC/AHA alertam que o uso precoce e isolado de corticoides está associado a um maior risco de recorrência. Por isso, recomendam doses baixas (como 0,2 a 0,5 mg/kg/dia) e desmame progressivo, preferencialmente com colchicina associada.^33,34^ A diretriz brasileira, entretanto, não especifica doses, tempo de uso nem estratégias de desmame, o que pode limitar sua aplicabilidade clínica em casos mais complexos.

Uma diferença notável é a ênfase da ACC/AHA na estratificação de risco. Ela propõe critérios objetivos para internação, como febre > 38 °C, derrames volumosos ou tamponamento, resposta inflamatória persistente após uma semana de tratamento e evidência de miocardite.^34^ A ESC menciona fatores prognósticos de forma mais ampla.

Além disso, a ACC/AHA e a ESC introduzem estratégias terapêuticas modernas, como o uso de bloqueadores de IL-1 (por exemplo, anakinra, rilonacept) e imunossupressores (como azatioprina) em casos refratários ou corticodependentes – recursos ainda não contemplados na diretriz brasileira.

Essa análise reforça a necessidade de atualização da diretriz nacional, que permanece sem revisão há mais de uma década e não incorpora abordagens modernas de estratificação de risco, duração terapêutica ou imunomodulação, já adotadas por sociedades internacionais.

Para pacientes com pericardite aguda refratária ao tratamento convencional, a terapia com imunossupressores, como a azatioprina ou o metotrexato, pode ser considerada em conjunto com outros medicamentos, embora essa abordagem necessite de mais estudos para definir seu papel exato e segurança.^33^ Em África, o ensaio randomizado controlado IMPI é uma referência, tendo sido o primeiro a investigar o papel dos esteroides na pericardite tuberculosa. Os resultados mostraram que, entre os pacientes HIV negativos com pericardite tuberculosa, a prednisolona reduziu a incidência de pericardite constritiva. No entanto, houve um aumento significativo na incidência de cancro associado ao HIV.^35^

Além da terapia farmacológica, repouso relativo também é frequentemente recomendado durante o período agudo da doença. A restrição de exercícios é fundamental como terapia para todos os pacientes durante os sintomas e por pelo menos 3 meses para atletas; o atleta pode retornar às atividades esportivas competitivas somente após os sintomas terem desaparecido e os testes diagnósticos (ou seja, proteína C-reativa, ECG e ecocardiograma) estiverem normalizados.^1,36,37^ Monitoramento próximo e avaliação regular são essenciais para controlar a pericardite aguda, especialmente em casos mais graves ou complicados.^33^ É fundamental ressaltar que o tratamento da pericardite aguda deve ser personalizado, considerando a gravidade dos sintomas, a presença de condições subjacentes e as características individuais do paciente. Diretrizes clínicas frequentemente atualizadas oferecem orientações precisas para garantir o manejo eficaz e seguro dessa condição.

Embora geralmente seja autolimitada, a pericardite pode levar à incapacidade significativa a curto prazo. Formas complexas da doença demandam atenção especial. As principais complicações a serem consideradas incluem: persistência dos sintomas, recorrência, tamponamento cardíaco (cerca de 1% dos casos) e constrição pericárdica.^7,33^ O tratamento das formas complexas da pericardite visa aliviar os sintomas, prevenir a recorrência e monitorar possíveis complicações. As principais medidas incluem o uso de AINEs e, em casos mais graves, podem ser utilizados corticoides ou colchicina, além da cardiovigilância por meio de ECG, ecocardiograma e exames laboratoriais.^7,12^ Cada caso de pericardite complexa deve ser avaliado individualmente por um cardiologista experiente, que irá definir o melhor plano de tratamento e acompanhamento, proporcionando ao paciente uma melhor qualidade de vida.^12,36^

### Perspectiva futura

Aprimorar o conhecimento fisiopatológico da pericardite aguda é essencial para explorar novas opções de tratamento, uma vez que a compreensão detalhada dos mecanismos subjacentes à doença pode identificar alvos terapêuticos específicos e abrir portas para intervenções mais eficazes (Figura Central). A investigação contínua sobre os processos inflamatórios e imunológicos envolvidos na pericardite aguda tem potencial para revolucionar o manejo clínico da condição. Um exemplo promissor é o tratamento inovador por meio da via de ação da IL-1, que evidencia como o avanço do conhecimento científico pode levar ao desenvolvimento de novas terapias. Este tratamento direcionado à IL-1 demonstra a capacidade de modular a resposta inflamatória, proporcionando uma abordagem mais precisa e eficaz para os pacientes, reduzindo os sintomas e prevenindo recorrências. Assim, a integração de novas descobertas científicas no campo da pericardite aguda pode transformar significativamente as estratégias terapêuticas, melhorando a qualidade de vida dos pacientes e otimizando os resultados clínicos.^38–40^

A melhor caracterização de casos subagudos é fundamental para identificar o tratamento mais adequado para esses pacientes. O sobrediagnóstico da pericardite aguda indica que diversos parâmetros, como o volume de pequenos derrames pericárdicos, podem não ser precisos para o diagnóstico. É, portanto, necessário aprimorar a estratificação do risco de agravamento da doença e utilizar novas ferramentas de diagnóstico, como a TC, o PET e a RMC. Multimodalidade em cardioimagem é extremamente útil para afastar casos que foram equivocadamente diagnosticados com pericardite aguda a partir de um derrame pericárdico discreto ou pequeno com uma dor típica não característica.^41,42^

A inteligência artificial (IA), especialmente por meio de algoritmos de aprendizado profundo (*deep learning*), tem emergido como uma ferramenta promissora para o diagnóstico de pericardite aguda, sobretudo no contexto da emergência, onde a diferenciação entre pericardite e infarto agudo do miocárdio com supradesnivelamento do segmento ST representa um desafio clínico relevante. A sobreposição de manifestações eletrocardiográficas entre essas condições pode levar a erros diagnósticos, com consequências terapêuticas graves. Nesse cenário, pesquisadores da Escola de Medicina do Centro Médico de Defesa Nacional de Taipé, em Taiwan, desenvolveram um modelo de aprendizado profundo baseado em ECG de 12 derivações que superou cardiologistas e algoritmos comerciais na detecção de pericardite aguda. O modelo atingiu sensibilidade de 78,9% e especificidade de 97,7%. Além da função diagnóstica, o modelo também demonstrou potencial prognóstico. Pacientes que foram falsamente classificados como pericardite, mas não atendiam aos critérios clínicos para tal, apresentaram risco significativamente maior de hospitalização por causas cardíacas em até três dias, sugerindo que o modelo pode identificar padrões eletrocardiográficos subclínicos preditores de eventos cardiovasculares.^43^

Apesar dos resultados promissores, limitações importantes ainda restringem a aplicabilidade clínica desses modelos. Entre elas estão: a necessidade de validação externa em amostras populacionais diversas, o caráter retrospectivo e unicêntrico do estudo original e o desafio da "caixa-preta" dos modelos de aprendizado profundo, cuja interpretação clínica ainda é limitada. Além disso, pacientes com pericardite crônica, tamponamento cardíaco ou outras condições com apresentações semelhantes foram excluídos do treinamento, o que pode comprometer a generalização do algoritmo para diferentes formas de manifestação da doença.

Ainda assim, a incorporação de IA ao diagnóstico de pericardite representa uma fronteira inovadora, com aplicabilidade futura em ambientes de emergência, em unidades remotas e até mesmo por meio de dispositivos vestíveis. Em conjunto com a telemedicina e o monitoramento remoto, esses sistemas poderão, futuramente, contribuir não apenas para o diagnóstico precoce, mas também para o acompanhamento longitudinal e para a estratificação de risco individualizada em pacientes com pericardite.

Instituições com o objetivo de defesa e informação sobre a pericardite, tanto para pacientes quanto para profissionais de saúde, têm sido cruciais no cenário atual, destacando-se a Pericarditis Alliance, fundada em 2020. Essa instituição é composta por pacientes que enfrentaram desafios significativos para obter um diagnóstico correto e um tratamento eficaz, unindo-se a prestigiados profissionais de saúde e pesquisadores na missão de avançar no diagnóstico e no tratamento da pericardite. O trabalho da Pericarditis Alliance abrange pesquisa, educação e apoio, áreas em que ainda há uma carência considerável de conscientização, investigação e advocacia sobre as causas, a prevenção e o tratamento da pericardite crônica, complicada ou recorrente.^44^ Instituições como a Pericarditis Alliance desempenham um papel fundamental na ampliação do conhecimento e na melhoria dos cuidados de saúde, especialmente em áreas subexploradas, como a pericardite. O crescimento contínuo destas organizações é fundamental para transformar o cenário do manejo da pericardite, contribuindo para um futuro com diagnósticos e tratamentos melhores.

## Conclusão

Em conclusão, o tratamento da pericardite aguda está em constante evolução, com uma variedade de opções terapêuticas disponíveis. O foco principal continua sendo o alívio dos sintomas, a prevenção de recorrências e de complicações, garantindo uma abordagem abrangente e personalizada para cada paciente. Novas pesquisas e descobertas certamente continuarão a aprimorar o manejo clínico desta condição.

## Data Availability

Os conteúdos subjacentes ao texto da pesquisa estão contidos no manuscrito.
