# Supplementary material for: Fundamentos da Pericardite Aguda: Estado da Arte
Source: Arq Bras Cardiol. 2026 Mar 26;123(2):e20240572. [Article in Portuguese] doi: 10.36660/abc.20240572 (PMC13128203; doi:10.36660/abc.20240572)
Supplement: Translation

# Translation: Fundamentals of Acute Pericarditis: State of the Art

**DOI:** 10.36660/abc.20240572i

**Authors:** Gabriel de Moraes Mangas, Jose Gregorio Valero Rodriguez, João Alexandre Ranzeiro de Bragança dos Santos, Flavia Nunes Benicio de Souza, Luis Felipe Leite da Silva, Giovane Leal de Azevedo, Alberto Chivaca, Neusa Perina de Jesus Jessen, Ângelo Oliveira, Evandro Tinoco Mesquita

**Affiliations:** 1 Niterói RJ Brazil Universidade Federal Fluminense, Niterói, RJ – Brazil; 2 Maputo Mozambique Hospital Central de Maputo, Maputo – Mozambique; 3 Maputo Mozambique Universidade Eduardo Mondlane, Maputo – Mozambique; 4 Niterói RJ Brazil Complexo Hospitalar de Niterói, Niterói, RJ – Brazil

**Keywords:** Acute Pericarditis, Inflammation, Diagnosis, Treatment, Artificial Intelligence

## Abstract

Acute pericarditis is an inflammation of the pericardium, the lining that surrounds the heart. It is the most common inflammatory heart condition. The disease frequently affects young adults and can manifest with varying severity. Pericarditis can have diverse causes, including viral infections, autoimmune diseases, post-infarction conditions, and, more recently, SARS-CoV-2 infections or COVID-19 vaccines. In developing countries, especially in Africa, tuberculosis is the leading cause of pericarditis, often associated with HIV. Diagnosis is based on clinical criteria, such as chest pain and electrocardiographic changes, and it can be supported by imaging studies such as echocardiography, cardiac magnetic resonance imaging, and computed tomography. Identification of etiology is crucial to personalized treatment, although the specific cause is often unidentified. New research has highlighted the role of the NLRP3 inflammasome in the pathophysiology of pericarditis, which may pave the way for new therapies. Furthermore, technological advancements and artificial intelligence are discussed as promising tools to improve the management of pericarditis.

## Introduction

Acute pericarditis is the most common inflammatory heart disease, with an estimated incidence of 27.7 per 100,000 people in the Western world.^1^ In the African continent, a systematic review of existing evidence showed that the prevalence of pericardial diseases varies greatly according to the population studied, with prevalences of around 1.1% found in individuals with cardiac complaints, between 3.3% and 6.8% in patients with heart failure, and up to 46.5% in HIV-infected patients with cardiac symptoms.^2^

Although it generally follows a benign and self-limiting course, pericarditis can present with severe symptoms. The condition frequently affects young adults, with frequent recurrence.^3,4^ Prognosis is associated with the patient's age and the etiology of pericarditis.^5,6^ Therefore, a multidisciplinary approach, involving collaboration between cardiologists, rheumatologists, oncologists, immunologists, and infectious disease specialists, may result in better patient outcomes.^7,8^ Advances in the pathophysiological understanding of acute pericarditis have revealed the central role of the NLRP3 inflammasome and the subsequent release of interleukins (IL-1α and IL-1β) in the development of the inflammatory response.^9,10^ This pathophysiological process has been the target of new therapeutic approaches to pericarditis, including the IL-1 receptor antagonist, rilonacept.^11^

Within this context, this review aims to highlight recent advances in treatment, diagnosis, prognostic factors, pathophysiology, and current guidelines regarding acute pericarditis. Furthermore, the review discusses the potential role of emerging technologies that use artificial intelligence in developing better management strategies for patients with pericarditis.

## Methods

A narrative review on pericarditis was conducted by means of a structured electronic search. The search strategy was designed using controlled descriptors (MeSH/DeCS) combined with Boolean operators (AND, OR), encompassing studies published in English, Portuguese, or Spanish from January 2010 to August 2024. Searches were performed in the following databases: MEDLINE/PubMed, Cochrane Library, LILACS/IBECS, and Embase. The search included primary studies, case reports, systematic reviews, narrative reviews, and qualitative studies. Additionally, relevant articles were identified through manual screening of the reference lists of selected studies and a supplementary web search to capture publications not located in the initial search. Publications that did not address the diagnosis or management of pericarditis were excluded. The following search terms were used: "Pericarditis"; "Pericardite"; "Acute Pericarditis"; "Pericardite Aguda"; "Inflammation"; "Inflamação"; "Diagnosis"; "Diagnóstico"; "Diagnostic Imaging"; "Imagem Diagnóstica"; "Therapeutics"; "Tratamento"; "Treatment"; "Anti-inflammatory agents"; "Follow-Up Studies"; "Acompanhamento"; "Monitoring"; "Long-term follow-up"; "Artificial Intelligence"; "Inteligência Artificial"; "Machine Learning"; "Aprendizado de Máquina". Given the nature of the study, as a narrative review that did not directly involve humans or animals, approval by an ethics committee was not required.

**Figure:** Conceptual map of the etiology, clinical presentation, diagnosis, and medical treatment of acute pericarditis. CT: computed tomography; IL-1: interleukin-1; MRI: magnetic resonance imaging; NSAIDs: non-steroidal anti-inflammatory drugs; PET-CT: positron emission tomography combined with computed tomography.

### Etiology and pathophysiology

The pericardial sac consists of two layers: the visceral and the parietal. The visceral layer, composed of pericardial mesothelial cells, adheres directly to the epicardium, whereas the more fibrous parietal layer provides structural support. Pericardial mesothelial cells are essential for the proper functioning of the heart. They play a crucial role in lubrication, protection, and regulation of inflammation within the pericardium. Alterations in mesothelial cell function can lead to several conditions, including pericarditis, pericardial effusion, and cardiac tamponade. Referred shoulder pain is due to somatosensory fibers of the phrenic nerve that innervate the parietal pericardium.^11^

Pericarditis is the most common form of pericardial disease and is defined as inflammation of the pericardial layers. It may be associated with pericardial effusion and result in cardiac tamponade. The disease can be an isolated cardiac manifestation or the result of a systemic disorder.

Pericarditis can have diverse etiologies, both infectious and non-infectious, or it can be idiopathic (Central Illustration).^12^ As illustrated in Table 1, pericarditis can be caused by infection (viral, bacterial, fungal, and parasitic), autoimmune diseases, metabolic disorders, myocardial infarction, trauma, post-pericardiotomy syndrome, neoplasms, radiotherapy, and medications.^13^ The most common etiologies are infectious causes, with viral pericarditis predominating (approximately 80% to 85% of cases).^1^ In this case, inflammation results from the direct action of the virus or the body's immune response to the agent.^14^ Furthermore, a clinical presentation of apparent recent viral infection (upper respiratory tract infection or gastroenteritis) has been documented in approximately 40% of patients with acute pericarditis.^1^

**Table 1:** Main etiological agents of acute pericarditis

Etiology	Details	Important observations
**Infectious**	Viral, bacterial, fungal, and parasitic	– 80% ato 85% of all cases are viral. – Approximately 40% of patients with acute pericarditis have a history of recent infection (upper respiratory tract or gastrointestinal tract) – Cases caused by bacterial infection may originate from pneumonia, rheumatic fever, empyema, hematogenous dissemination, or post-cardiac/thoracic surgery. – In developing countries, 70% to 80% of cases are due to tuberculosis. – HIV infection is a risk factor.
**Autoimmune diseases**	Systemic lupus erythematosus, rheumatoid arthritis, scleroderma, polymyositis, and dermatomyositis	
**Metabolic disorders**	Hypothyroidism, hyperlipidemia, and diabetes mellitus	Metabolic disorders indirectly affect the pericardium through inflammatory reactions or metabolites that are toxic to pericardial cells.
**Acute myocardial infarction**	Inflammatory reaction	It may occur early (3 days afterwards), known as peri-infarction pericarditis, or late (3 weeks to 6 months) due to autoimmunity, known as Dressler syndrome.
**Trauma**	External object	Mainly in the thorax
**Post-pericardiotomy syndrome**	Local or systemic inflammatory reaction	This etiology has become more common with an increasing number of interventions.
**Neoplasms**	Tumor or lymphatic invasion	Hematogenous dissemination
**Radiotherapy**	Direct tissue damage (pericardial cells), inflammatory reaction, fibrosis, and vascular damage	Mainly in thoracic cancer, especially breast, lung, and lymphoma
**Medications**	Cardiotoxicity due to radiotherapy or chemotherapy	Immune checkpoint inhibitors are recently used drugs that may be associated with pericarditis.
**Kidney failure**	Uremic pericarditis or pericarditis associated with dialysis	Approximately 20% of cases progress to pericardial effusion.

Furthermore, local epidemiological data should always be considered when defining etiology.^1^ Based on evidence from developed countries, the most prevalent etiological agents are viruses, and cases of acute pericarditis are frequently preceded by a flu-like or gastrointestinal syndrome. An increase in cases of acute pericarditis is also observed during colder seasons. In a prospective study in France, 55% of cases were of idiopathic origin, and 20% followed a heart injury syndrome. The latter cause is progressively increasing in developed countries due to the growing number of cardiac procedures.^12^ Post-infarction pericarditis can occur early, within the first 3 days after the acute myocardial infarction episode, which is known as peri-infarction pericarditis, or late (from 3 weeks to 6 months afterwards), due to autoimmune activity, which is known as Dressler syndrome.

Moreover, pericarditis in people living with HIV can be due to infectious, non-infectious, or neoplastic diseases (Kaposi's sarcoma or lymphoma), which can result in myopericarditis. In cases of autoimmune etiology, pericarditis occurs mainly in cases of systemic lupus erythematosus, rheumatoid arthritis, scleroderma, polymyositis, and dermatomyositis. Another common cause of pericardial disease is kidney failure, which progresses to the development of effusion in 20% of cases, and may manifest as uremic pericarditis or dialysis-associated pericarditis.^14^

Other possible causes to be considered are hypothyroidism and cancer. Neoplastic pericarditis is a consequence of tumor or lymphatic invasion, or it occurs through hematogenous dissemination, thus occurring in both primary and metastatic forms, or even as a result of radiotherapy for thoracic cancers. There are currently only a few cases of pericarditis due to immune checkpoint inhibitors described in the literature.^3,12^ Another mechanism associated with acute pericarditis in oncology patients involves cardiotoxicity resulting from radiotherapy drugs and, more recently, immune checkpoint inhibitors.^12^

In contrast, when the etiology is bacterial, pericarditis normally presents with pericardial effusion, and its origin may be related to situations such as pneumonia, rheumatic fever, empyema, hematogenous dissemination, or the postoperative period following cardiac or thoracic surgery.^14^

In developing countries, the high prevalence of infectious diseases such as HIV, associated with poverty and weak existing health systems, contributes to infectious diseases playing an important role in the spectrum of heart failure etiology. An analysis of studies that assessed the etiology of heart failure in several sub-Saharan African countries found pericarditis in 3% of cases.^15^ In the Sub-Saharan Africa Survey of Heart Failure (THESUS–HF) study, which included 1,006 patients with heart failure from 9 African countries, 6.8% presented with pericardial effusion with tamponade.^16^ It is also noteworthy that, in developing countries, the main etiology of pericarditis is tuberculosis, reaching 70% to 80% of cases or 90% among HIV-positive patients.^17^

In Africa in particular, a systematic review of the available evidence showed that few studies reported on the etiology of pericardial disease, and most of them included a small sample of patients.^18^ Among published studies, tuberculosis was the most frequently observed cause of pericardial disease, and HIV infection was the main risk factor; however, tuberculosis was also the main etiology in patients not infected with HIV. An echocardiographic-based study conducted at a tertiary hospital in Dar es Salaam assessed the pattern of cardiac anomalies found in HIV-infected patients presenting with cardiac symptoms. The most common echocardiographic diagnosis was pericardial effusion, present in 41.2% of study participants.^19^ However, in most reported cases, the etiology of pericardial disease was not found, and in the remaining cases, neoplasms and systemic diseases such as systemic lupus erythematosus were the most frequently found etiologies.^18^

In addition to the most widely known etiologies of acute pericarditis, two important new etiologies have been described in the past 2 years, namely, SARS-CoV-2 infection and the COVID-19 vaccine, especially the mRNA vaccine. With these two new etiologies, pericarditis that occurs in association with myocarditis has gained more relevance. Pericarditis was present in 1.5% of COVID-19 cases, and it is more common in older individuals. Although it is a possible etiology of pericarditis, current data show that the benefits of vaccination against respiratory infections outweigh concerns about possible, but definitely rare, vaccine-induced pericarditis. Furthermore, the clinical course of pericarditis due to this etiology does not differ when compared to the common presentation of viral pericarditis.^20,21^

During the diagnostic period of pericarditis, viral infection cannot be confirmed by antibody serology, given that IgM antibodies are no longer detectable. Molecular techniques, such as polymerase chain reaction (PCR) tests performed on pericardial fluid or tissue after pericardiocentesis or pericardial biopsy, respectively, could identify the etiological agent, but this is an invasive approach, and it is not recommended for a disorder that generally follows a benign course. According to recent guidelines, routine identification of the viral agent is not recommended, as it does not affect treatment decisions and prognosis, with the exception of hepatitis C virus and HIV. The terms viral and idiopathic pericarditis are treated synonymously, presuming a viral etiology in the idiopathic form. In summary, identification of the underlying etiology is of paramount importance for developing a personalized treatment plan for pericarditis, and it is recommended that pericarditis be investigated in patients with systemic diseases.^1^

Regarding molecular and cellular pathophysiology, new clinical trials in rodents have shown that the NLRP1 (NACHT, leucine-rich repeat, and pyrin domain 1) inflammasome and IL-1β play an important role in the development of acute pericarditis inflammation and its recurrence.^22^ Inflammasomes are platforms composed of proteins that control the inflammatory response and coordinate the body's antimicrobial defenses. They are activated by the detection of pathogenic microorganisms and danger signals in the cytoplasm of cells, activating caspase-1, which produces cytokines and induces pyroptosis of cells through the activation of gasdermin D. Exacerbated activation of inflammasomes is related to numerous hereditary and acquired inflammatory disorders, such as pericarditis.^13^ This evidence should guide further studies aimed at investigating the mechanism of development of pericarditis, which often has no defined etiology and is therefore termed idiopathic.^22^

### Diagnosis

As shown in the Central Illustration, diagnosis of acute pericarditis should include at least two of the following criteria: pericardial pain, pericardial friction rub, new ST-segment elevation or PR-segment depression on electrocardiography (ECG), or the appearance of a new or worsening pericardial effusion (demonstrated by a cardiac imaging method).^12^ Pericardial pain is acute and retrosternal; it typically worsens with inspiration, coughing, or lying down and improves when the patient sits upright or leans forward.^11^ It may also radiate to the top of the scapulae, arms, neck, or jaw.^12,23^ Upon physical examination, in addition to pericardial friction rub, which may be auscultated at the left sternal border, low-grade fever and other manifestations may be found if the underlying disease is systemically present. There is no specific biomarker for pericarditis; however, biomarkers of inflammation, such as C-reactive protein, are increased in 80% of cases, and troponin may be mildly elevated if the inflammation has reached the myocardium, defining myopericarditis.^12^

On the other hand, etiological diagnosis is challenging, and in many cases the cause of pericarditis remains undetermined. Even in the specific context of suspected tuberculous pericarditis, definitive diagnosis has shown to be difficult, prompting studies on the accuracy of new diagnostic tests, including the quantitative PCR test (Xpert MTB/RIF), unstimulated interferon gamma (uIFNγ), pericardial adenosine deaminase (ADA), and pericardial and urinary lipoarabinomannan (LAM). In a comparative study between Xpert MTB/RIF, pericardial ADA, and uIFNγ, the uIFNγ assay demonstrated the highest diagnostic accuracy and may serve as the first-line test for the diagnosis of tuberculous pericarditis. Additionally, greater sensitivity and specificity are achieved by combining Xpert MTB/RIF with either ADA or uIFNγ.^24^ Conversely, urinary or pericardial LAM showed low sensitivity but high specificity for diagnosis of tuberculous pericarditis, and urinary LAM showed greater sensitivity in HIV-infected patients with CD4 ≤ 100 cells/mm^3^.^25^ However, access to these diagnostic tests poses another important challenge in African countries, where tuberculosis plays a more important role in the etiology of pericarditis. Therefore, when pericardial fluid can be collected, fluid culture remains the most widely used diagnostic test, and ADA remains the most widely used biochemical test. Nonetheless, culture requires at least 3 weeks to yield results, and ADA has limited value as a confirmatory diagnostic test. Moreover, in regions with high tuberculosis endemicity, a positive tuberculin skin test only reflects exposure to *Mycobacterium tuberculosis* antigens and does not accurately discern active disease, thus limiting its value.^26^

Imaging studies can be useful in the diagnosis and follow-up of acute pericarditis, especially echocardiography, cardiac magnetic resonance imaging (MRI), and computed tomography (CT), as shown in Figure 1.^23^ In the absence of a pericardial effusion with sufficient volume, conventional radiography normally shows no abnormalities.^11^

**Figure 1:** A, B) Apical four-chamber and parasternal short-axis echocardiographic views, respectively, showing the presence of effusion and significant fibrin in the pericardial space (arrows). C, D, E, F) Magnetic resonance imaging showing extensive non-ischemic fibrosis in the mesocardial and subepicardial regions.

Echocardiography should be the first-choice imaging modality, as it is an accessible, low-cost, and quick method capable of providing essential information about pericardial thickness, the presence and volume of pericardial effusion, and its hemodynamic impact on cardiac filling (pericardial tamponade). Echocardiography can also identify masses adherent to the pericardial cavity, fibrin strands, content with density suggestive of thrombus in pericardial fluid, and localized forms of effusion, as frequently observed in the postoperative period following cardiac surgery (Figure 2). Echocardiography may be normal, leading to a diagnosis of dry pericarditis, which may be present in up to 30% of patients. Additionally, small pericardial effusions can occur in healthy individuals; therefore, diagnosis of pericarditis should be made with caution. Although some patients with acute pericarditis present with normal echocardiograms, this examination is highly useful for diagnosing the presence and size of pericardial effusion, tamponade physiology, and myocardial involvement, as in cases of myopericarditis.^17^

**Figure 2:** Medical treatment regimen for pericarditis.

Tuberculous pericarditis, for example, usually presents with effusion, often in tamponade, and sometimes already in the constrictive pericarditis phase; it may also present as myopericarditis (Table 1). The important Investigation of the Management of Pericarditis in Africa (IMPI Africa) registry recruited 185 patients from 15 hospitals in three sub-Saharan African countries who were in the initial stages of treatment for tuberculous pericarditis. Of these, 79.5% presented with effusive pericarditis, 15.1% with effusive-constrictive pericarditis, and 5.4% with constrictive or acute dry pericarditis, and 40% of all patients presented with clinical characteristics of HIV infection.^27^

If echocardiography findings are inconclusive, clinical presentation is atypical, or the physician wishes to investigate specific causes such as malignancy or tuberculosis, CT or cardiac MRI may be used. On CT, we can observe pericardial thickness, calcification, and regional effusions. The density of pericardial fluid allows differentiation between transudate, exudate, blood, or, occasionally, the presence of a mass adjacent to the pericardium, such as lung or breast tumors. A particularly useful resource in cardiac MRI is late gadolinium enhancement (LGE), a technique that enables detection of pericardial inflammation with a sensitivity of 94% to 100%.^12,17^

PET-CT with 18F-FDG is an examination that is more commonly used in the context of oncology; however, it has proven useful to the extent that, in the context of acute pericarditis with pericardial effusion, greater absorption of FDG by the pericardium is associated with a higher risk of recurrence and may be useful in characterizing recurrent pericarditis.^17^

### Differential diagnosis

Differential diagnosis of acute pericarditis is essential due to its overlapping symptoms with various cardiac and non-cardiac conditions. Sudden chest pain is a common, but nonspecific symptom, and it can be caused by several conditions, including myocardial infarction, pulmonary embolism, aortic dissection, as well as gastrointestinal and musculoskeletal disorders. Initial assessment includes detailed physical examination and analysis of the patient's symptoms. Laboratory tests, such as troponin and C-reactive protein levels, are useful in differentiating acute pericarditis from myocardial infarction.^12,13^ The ECG frequently reveals diffuse ST-segment elevation, PR depression, and the ECG stages of acute pericarditis, a classic, though nonspecific, finding of the disease. Pericarditis is classically associated with ECG changes that evolve in four stages (Table 2).^28^

**Table 2:** Electrocardiogram changes according to progression of pericarditis

Stages	Electrocardiogram changes
**Stage 1**	Diffuse ST elevation and PR depression with reciprocal aVR changes (occurs during the first 2 weeks)
**Stage 2**	Normalization of ST-segment changes; generalized T-wave flattening (1 to 3 weeks)
**Stage 3**	T waves become inverted (3 to several weeks)
**Stage 4**	Electrocardiogram returns to normal (several weeks onward)

Chest radiography may show an enlarged heart or associated pleural effusion. Echocardiography is a valuable tool for assessing the presence of pericardial effusion and changes in cardiac function, but it may not be conclusive on its own. In cases of persistent suspicion or when the diagnosis is unclear, cardiac MRI or CT may be useful to evaluate cardiac structures in more detail. Excluding myocardial infarction is one of the main challenges in differential diagnosis. In patients with suspected acute pericarditis and risk factors for coronary artery disease, coronary angiography may be necessary to rule out coronary obstruction.

Moreover, it is important to consider the differential diagnosis of epipericardial fat necrosis (EFN). As with acute pericarditis, EFN may present with chest pain as the main symptom, which can make accurate diagnosis difficult. Therefore, it is important to assess the differences in pain location; in EFN, it is generally more pleuritic and located in the precordial region, whereas the pain in pericarditis may be more diffuse and radiate to the neck, shoulders, and back, with ECG changes. EFN generally does not alter ECG. Conversely, pericarditis may present with changes such as ST elevation and PR depression, as well as fever, fatigue, and pericardial friction rub, which are less frequent in EFN. CT is the examination of choice for diagnosing EFN, as it identifies areas of fat inflammation surrounding the heart. MRI and laboratory tests can also be helpful.^29,30^

It is important to consider other conditions that can mimic acute pericarditis, such as myocarditis, pulmonary embolism, and gastrointestinal diseases. Each of these conditions presents distinct clinical characteristics, and specific tests aid in accurate differential diagnosis. Careful diagnostic assessment and consideration of a variety of conditions that may manifest in a similar fashion to acute pericarditis are crucial for effective and accurate clinical management. The integration of clinical, laboratory, and imaging tests plays a fundamental role in the differential diagnostic approach to these cases.

Finally, the use of inadequate diagnostic criteria can lead to overdiagnosis of acute pericarditis. Small-volume pericardial effusion, chest pain, and elevated biomarkers may also be found in healthy individuals, and they do not necessarily indicate the presence of disease. Overdiagnosis of acute pericarditis can lead to unnecessary treatments, such as the administration of anti-inflammatory drugs or corticosteroids, which can impair the patient's quality of life and increase costs for the healthcare system.^12,13^

### Treatment

The main guidelines on pericarditis, ESC (2015), ACC/AHA (2020), and SBC (2013), show similarities regarding first-line treatment, but differ in important aspects regarding approach and risk stratification (Table 3).^14,31,32^

**Table 3:** Comparison between ESC (2015), ACC/AHA (2020), and SBC (2013) guidelines for treatment of acute pericarditis

Aspect	ESC (2015)	ACC/AHA (2020)	SBC (2013)
**NSAIDs**	Ibuprofen 600 to 800 mg 3 times/day or aspirin 750 to 1000 mg 3 times/day, for 1 to 2 weeks, with gradual weaning	Ibuprofen, aspirin, or indomethacin for 2 to 4 weeks, with gradual weaning after remission of symptoms	Ibuprofen 600 mg 3 times/day or aspirin 1000 mg 3 times/day for 1 to 2 weeks, with progressive reduction
**Colchicine** (acute episode)	0.5 to 1.0 mg/day for 3 months, adjusted according to weight and tolerance	0.5 to 1.0 mg/day for 3 months, with dose adjustment for weight	Class IIa recommendation, evidence B. Dose: 1 mg/day (0.5 mg twice daily) for 3 months. Not universally indicated
**Colchicine (recurring)**	0.5 to 1.0 mg/day for 6 months	0.5 to 1.0 mg/day for 6 months, adjusted for weight	Recommended for 6 months in cases of recurrent pericarditis
**Avoiding physical activity**	Not specified	Recommends avoiding physical exertion during treatment	Recommends clinical rest is recommended, without an objective definition of time or intensity
**Corticosteroids: indication**	Reserved for contraindication or failure to treat with NSAIDs/colchicine, autoimmune or uremic pericarditis	Reserved for contraindication or failure to treat with NSAIDs/colchicine, or pericarditis associated with specific diseases	Recommended in cases refractory to NSAIDs/colchicine, autoimmune or uremic pericarditis
**Corticosteroids:** dose and duration	Prednisone 0.2 to 0.5 mg/kg/day, with slow weaning and concomitant use with colchicine	Prednisone or equivalent at a low dose (0.2 to 0.5 mg/kg/day), with gradual tapering and association with colchicine	Prednisone 1 mg/kg/day for 2 weeks, with weaning at 4 to 6 weeks and association with colchicine
**Risk of recurrence with corticosteroids**	Early and isolated use associated with greater risk of recurrence	Early and isolated use associated with greater risk of recurrence	Isolated use can increase risk of recurrence; recommends avoiding
**Immunobiological agents** (IL-1 inhibitors)	Not specified	Recommended in refractory or corticosteroid-dependent cases (e.g., anakinra, rilonacept)	Not mentioned
**Hospitalization criteria**	Considers factors such as fever, large effusion, and myopericarditis as associated with worse prognosis	Defines objective criteria: fever > 38 °C, large effusion, treatment failure after 7 days, myocarditis	Indicates hospitalization in cases of tamponade, high fever, important effusion, or severe etiology

IL-1: interleukin-1; NSAIDs: non-steroidal anti-inflammatory drugs.

All three recommend the initial use of non-steroidal anti-inflammatory drugs (NSAIDs) combined with colchicine. The ESC proposes the use of NSAIDs for 1 to 2 weeks, with gradual weaning, and colchicine for 3 months in the first episode and 6 months in relapses, at the usual dose of 0.5 to 1.0 mg/day, adjusted according to weight and tolerance (Figure 2). The ACC/AHA reinforces the same colchicine regimen, adjusted for body weight, and emphasizes the avoidance of intense physical activity during treatment. Both guidelines and recent articles highlight that the early addition of colchicine significantly reduces recurrence and prevents complications.^31–34^

According to all three guidelines, the use of corticosteroids, such as prednisone or prednisolone, is reserved for cases with contraindication or failure in the use of NSAIDs and colchicine or for pericarditis of autoimmune or uremic etiology. The ESC and ACC/AHA warn that the early and isolated use of corticosteroids is associated with a higher risk of recurrence. Therefore, they recommend low doses (such as 0.2 to 0.5 mg/kg/day) and progressive weaning, preferably with associated colchicine.^33,34^ The Brazilian guideline, however, does not specify doses, duration of use, or weaning strategies, which may limit its clinical applicability in more complex cases.

A noteworthy difference is the ACC/AHA guideline's emphasis on risk stratification. It proposes objective criteria for hospitalization, including fever > 38 °C, large effusions or tamponade, persistent inflammatory response after 1 week of treatment, and evidence of myocarditis.^34^ The ESC mentions prognostic factors more broadly.

Moreover, both the ACC/AHA and the ESC introduce modern therapeutic strategies, including the use of IL-1 blockers (e.g., anakinra, rilonacept) and immunosuppressants (e.g., azathioprine) in refractory or corticosteroid-dependent cases; these resources have yet to be included in the Brazilian guideline.

This analysis reinforces the need to update the Brazilian guideline, which has remained unrevised for more than a decade and does not incorporate modern approaches to risk stratification, therapeutic duration, or immunomodulation, which have already been adopted by international societies.

For patients with acute pericarditis refractory to conventional treatment, immunosuppressive therapy, such as azathioprine or methotrexate, may be considered in conjunction with other medications, although this approach requires further studies to define its exact role and safety.^33^ In Africa, the IMPI randomized controlled trial is a reference as the first to investigate the role of steroids in tuberculous pericarditis. The results showed that, among HIV-negative patients with tuberculous pericarditis, prednisolone reduced the incidence of constrictive pericarditis. However, there was a significant increase in the incidence of HIV-associated cancer.^35^

In addition to pharmacological therapy, relative rest is also frequently recommended during the acute phase. Exercise restriction is essential as therapy during symptoms in all patients and for at least 3 months in athletes; athletes may return to competitive sports activities only once symptoms have disappeared and diagnostic tests (i.e., C-reactive protein, ECG, and echocardiogram) are normal.^1,36,37^ Close monitoring and regular assessment are essential to control acute pericarditis, especially in more severe or complicated cases.^33^ It is crucial to emphasize that the treatment of acute pericarditis must be personalized, considering the severity of symptoms, the presence of underlying conditions, and individual patient characteristics. Frequently updated clinical guidelines offer precise guidance to ensure the effective and safe management of this condition.

Even though generally self-limiting, pericarditis can lead to significant short-term disability. Complex forms of the disease require special attention. The main complications to be considered include persistent symptoms, recurrence, cardiac tamponade (approximately 1% of cases), and pericardial constriction.^7,33^ Treatment of complex forms of pericarditis aims to relieve symptoms, prevent recurrence, and monitor possible complications. The main measures include use of NSAIDs; in more severe cases, corticosteroids or colchicine may be used, in addition to cardiac monitoring through ECG, echocardiogram, and laboratory tests.^7,12^ Each case of complex pericarditis should be individually assessed by an experienced cardiologist, who will define the optimal treatment and follow-up plan, thus promoting better patient quality of life.^12,36^

### Future perspectives

It is essential to enhance pathophysiological knowledge of acute pericarditis in order to explore new treatment options, given that detailed understanding of the disease's underlying mechanisms can identify specific therapeutic targets and open doors to more effective interventions (Central Illustration). Ongoing investigation into the inflammatory and immunological processes involved in acute pericarditis has the potential to revolutionize the clinical management of the condition. A promising example is innovative treatment through the IL-1 pathway of action, which illustrates how advances in scientific knowledge can lead to the development of new therapies. This IL-1–targeted treatment demonstrates the ability to modulate the inflammatory response, providing a more precise and effective approach for patients, reducing symptoms, and preventing recurrences. Thus, the integration of new scientific discoveries in the field of acute pericarditis can significantly transform therapeutic strategies, improving patient quality of life and optimizing clinical outcomes.^38–40^

Better characterization of subacute cases is fundamental in order to identify the most appropriate treatment for these patients. Overdiagnosis of acute pericarditis indicates that several parameters, for example, the volume of small pericardial effusions, may not be accurate for diagnosis. Therefore, it is necessary to improve the risk stratification of disease progression and employ new diagnostic tools, including CT, PET, and cardiac MRI. Multimodality in cardiac imaging is extremely useful in ruling out cases that have been mistakenly diagnosed with acute pericarditis based on a discrete or small pericardial effusion with typical, non-characteristic pain.^41,42^

Artificial intelligence, especially deep learning algorithms, has emerged as a promising tool for diagnosis of acute pericarditis, particularly in emergency settings, where differentiating between pericarditis and ST-segment elevation acute myocardial infarction represents a significant clinical challenge. Overlapping ECG manifestations between these conditions can lead to diagnostic errors with severe therapeutic consequences. In this scenario, researchers at the School of Medicine of the National Defense Medical Center in Taipei, Taiwan, developed a 12-lead ECG-based deep-learning model that outperformed cardiologists and commercial algorithms in detecting acute pericarditis. The model achieved a sensitivity of 78.9% and a specificity of 97.7%. In addition to its diagnostic function, the model also demonstrated prognostic potential. Patients who were falsely classified as having pericarditis, but did not meet the clinical criteria for it, had a significantly higher risk of hospitalization for cardiac causes within 3 days, suggesting that the model can identify subclinical ECG patterns that predict cardiovascular events.^43^

Despite promising results, important limitations still restrict the clinical applicability of these models, including the following: the need for external validation in diverse population samples, the retrospective and single-center nature of the original study, and the "black box" challenge of deep learning models, whose clinical interpretation remains limited. Furthermore, patients with chronic pericarditis, cardiac tamponade, or other conditions with similar presentations were excluded from the training, which may compromise the generalization of the algorithm to different forms of disease manifestation.

Nonetheless, the incorporation of artificial intelligence into the diagnosis of pericarditis represents an innovative frontier, with future applicability in emergency settings, remote units, and even by means of wearable devices. In conjunction with telemedicine and remote monitoring, in the future, these systems may contribute not only to early diagnosis but also to longitudinal follow-up and individualized risk stratification in patients with pericarditis.

Institutions with the goal of advocating for and providing information about pericarditis, both for patients and healthcare professionals, have been crucial in the current scenario, especially the Pericarditis Alliance, founded in 2020. The institution is composed of patients who have faced significant challenges in obtaining a correct diagnosis and effective treatment, joining prestigious healthcare professionals and researchers in the mission of advancing the diagnosis and treatment of pericarditis. The Pericarditis Alliance's work encompasses research, education, and support, areas where there is still a considerable lack of awareness, investigation, and advocacy regarding the causes, prevention, and treatment of chronic, complicated, or recurrent pericarditis.^44^ Institutions like the Pericarditis Alliance play a fundamental role in expanding knowledge and improving healthcare, especially in underexplored areas such as pericarditis. The continuing growth of these organizations is crucial to transforming the landscape of pericarditis management, contributing to a future with better diagnoses and treatments.

## Conclusion

In conclusion, the treatment of acute pericarditis is constantly evolving, with a variety of therapeutic options available. The main focus remains on symptom relief and prevention of recurrences and complications, ensuring a comprehensive and personalized approach for each patient. Further research and discoveries will certainly continue to enhance the clinical management of this condition.

## Data Availability

The underlying content of the research text is contained within the manuscript.
